# Purification Analysis, Intracellular Tracking, and Colocalization of Extracellular Vesicles Using Atomic Force and 3D Single-Molecule Localization Microscopy

**DOI:** 10.1021/acs.analchem.3c00144

**Published:** 2023-03-31

**Authors:** Sujitha Puthukodan, Martina Hofmann, Mario Mairhofer, Hannah Janout, Jonas Schurr, Fabian Hauser, Christoph Naderer, Johannes Preiner, Stephan Winkler, Dmitry Sivun, Jaroslaw Jacak

**Affiliations:** ∇ University of Applied Sciences Upper Austria, Linz 4020, Austria; ∥ University of Applied Sciences Upper Austria, Hagenberg 4232, Austria; ⊥ Department of Computer Science, Johannes Kepler University, Linz 4040, Austria; † AUVA Research Center, Ludwig Boltzmann Institute for Experimental and Clinical Traumatology, Vienna 1200, Austria

## Abstract

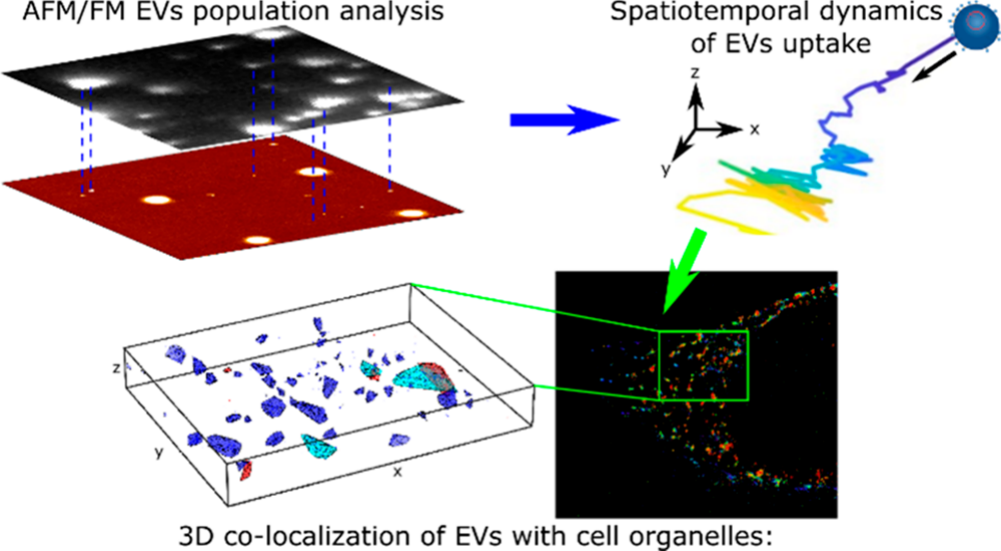

Extracellular vesicles (EVs) play a key role in cell–cell communication and thus have great potential to be utilized as therapeutic agents and diagnostic tools. In this study, we implemented single-molecule microscopy techniques as a toolbox for a comprehensive characterization as well as measurement of the cellular uptake of HEK293T cell-derived EVs (eGFP-labeled) in HeLa cells. A combination of fluorescence and atomic force microscopy revealed a fraction of 68% fluorescently labeled EVs with an average size of ∼45 nm. Two-color single-molecule fluorescence microscopy analysis elucidated the 3D dynamics of EVs entering HeLa cells. 3D colocalization analysis of two-color direct stochastic optical reconstruction microscopy (dSTORM) images revealed that 25% of EVs that experienced uptake colocalized with transferrin, which has been linked to early recycling of endosomes and clathrin-mediated endocytosis. The localization analysis was combined with stepwise photobleaching, providing a comparison of protein aggregation outside and inside the cells.

## Introduction

Extracellular vesicles (EVs) are nanometer-sized, lipid bilayer-enclosed particles which are released by cells and contain lipids, metabolites, proteins, and nucleic acids.^1−4^ They contribute to numerous cellular processes, including physiological and pathophysiological intercellular communications, immunomodulation, and inflammation, and also play roles in cancer and neurodegeneration.^3,5−7^ Thus, they have great potential for a host of clinical applications as diagnostic tools to study disease progression,^8,9^ therapeutic applications such as drug delivery,^10−13^ and regenerative medicines.^14,15^

The two major types of EVs are ectosomes (including microvesicles) released directly from the cell surface and endosome-derived vesicles (including exosomes) which are released when multivesicular bodies fuse with the cell membrane.^16,17^ The small fraction of endosome-derived (small) EVs (sEVs) are 30–100 nm in size and enriched with proteins such as CD9, CD63, and CD81 which belong to the tetraspanin family and are often employed as EV markers.^18,19^ Recently, various mechanisms have been proposed for cellular internalization of EVs: clathrin-mediated endocytosis, caveolin-dependent endocytosis, micropinocytosis, phagocytosis, and receptor-mediated endocytosis.^20−22^ So far none of these mechanisms have been studied extensively, in part due to a lack of precision tools. Methodological solutions which provide the capabilities to decipher the EVs’ population heterogeneity in terms of size and purity as well as to monitor the cellular uptake (dynamics) and colocalize the EVs to organelles need to be developed.

Observation of molecular processes is essential for understanding the function of biological systems. Deciphering the kinetics, transport, and localization of individual molecules is specifically important as it often uncovers mechanisms veiled in ensemble experiments.^23^ Fluorescence microscopy (FM) comes with the advantage of noninvasive imaging, and higher specificities have aided numerous cellular and molecular studies, including investigations of EVs.^17,24^ 3D single-molecule tracking helped to reveal intercellular transport of transferrin;^25^ it also was used to investigate spatiotemporal transport dynamics of endocytic nanoparticles in cells.^25^ Two-color quantum-dot tracking was used for quantification of receptor dimerization rates.^26^ There have been also numerous methodological advances in the study of EVs, particularly in relation to their uptake and internalization.^27−30^ Aggregation analysis can provide insights on the predominant up-take pathway.^20,31,32^ Studies using single-molecule localization microscopy (SMLM) allow for detailed analysis down to the single-EV level.^17,33−36^ Recently, atomic force microscopy (AFM) has gained importance for nanoscopic analysis of EVs as a complementary technology to SMLM.^37−40^ AFM has been used for quantitative analyses of their size, mechanical properties, and aggregation.^40,41^ Combining both of these single-molecule sensitive methods provides a unique, exhaustive toolbox for quantitative EV analyses.^42,43^

In this paper, we present a comprehensive characterization of the cellular uptake of EVs at the single-molecule level, for the first-time providing thorough information on the EV population and later on the up-take dynamics and colocalization to cell organelles of EVs from this population. As a model system, small EVs isolated from transfected HEK293T cells were used.^44^ To track individual EVs, the N-terminus of the tetraspanin CD63 protein was labeled with a green fluorescent protein (eGFP-CD63 EVs). We developed a toolbox for single-molecule sensitive analysis capable of quantification on aggregation, kinetics, and the EV sample quality using minimal sample amounts, which enabled measurements to decipher cellular uptake and biological efficacy. The combination of AFM and 2D single-molecule FM (SMFM) as well as 3D SMLM yielded a full picture of the purified, fluorescently labeled, endosome-derived EV population in terms of size, Young’s modulus, and number of eGFPs per EV.

The uptake of these EVs was previously confirmed in HeLa cells; they remain mainly in the cytosol and in proximity to the nucleus.^44^ Nevertheless, no information on the uptake dynamics of EVs is yet available. Therefore, we have carried out two-color 3D SMFM to simultaneously track EVs and visualize the cell membrane. The determined trajectories enabled deciphering a variety of EV diffusion behavior inside and outside the cell. In a post-uptake showcase study using two-color 3D localization microscopy and a newly developed software, we measured for the first time the colocalization of EVs with transferrin, which has been shown to be involved in early endosomes and recycling in cells.^45,46^ Accordingly, stepwise photobleaching of the signals originating from inside the cells was carried out and compared to the respective EV signals outside of the cells, providing information on EV-clustering inside the cells.

## Material and Methods

### Transfection, Cell Culture, Isolation, and Characterization of EVs

HEK293T cells (ATCC #CRL-3216) were cultured in Dulbecco’s Modified Eagles Medium (DMEM; Thermo Fisher, Waltham, MA, USA), high glucose, supplemented with 10% fetal bovine serum (FBS; Thermo Fisher, Waltham, MA, USA) and 1% penicillin/streptomycin (complete growth medium) at 37 °C in a 5% CO_2_ atmosphere. Cells were transfected with the eGFP CD63 plasmid (Addgene #62964) as described in ref (44). eGFP-CD63 EVs were isolated by differential ultracentrifugation and characterized by Western blotting.^44^ The pellet after centrifugation at 110 000*g* was stored in 40 μL of PBS at −80 °C and diluted for the experiments 1:100 with PBS.

### Single-Molecule Fluorescence Microscopy

Fluorescence imaging of EVs and HeLa cells was done in three dimensions by implementing 3D single-molecule imaging using astigmatism.^47^ A cylindrical lens (*f* = 1000 mm, Thorlabs) was introduced into the optical detection path to generate different focal planes for the *x* and *y* directions. 2D Gaussian elliptical fitting enables determining the *x*,*y*-elongation and consequently the *z*-position. A calibration curve of widths of *x* and *y* as a function of *z* were experimentally generated before each experiment using multiple colored fluorescent nanobeads (1:1000 μL, TetraSpeck, Thermo Fisher Scientific) immobilized on a glass substrate. These calibration curves also allowed for correction of any spectral aberration existing in the instrument. A stack of 200 frames with a step size of 10 nm over a range of 2 μm was acquired (20 ms illumination time, 33 ms delay). The calibration curve was calculated and 3D dSTORM images were analyzed using a custom written software in Qt/C++.^48^ Images were acquired using a modified Olympus IX81 inverted epifluorescence microscope with an oil-immersion objective (UApo N 60×/1.49 NA, Olympus). The sample was positioned on a three axes piezo stage (P-733.3DD, Physical Instruments) with nanometer precision on top of a mechanical stage with a range of 1 × 1 cm. The sample was illuminated by a 488 nm diode laser (Toptica Photonics, Graefelfing, Germany) in wide-field configuration. The cell membrane labeled with Alexa 647 conjugated anti-CD59 antibody (Thermo Fisher, Waltham, MA, USA) and a transferrin labeled with Alexa 647 were excited with a 646 nm diode laser (Toptica, Photonics, Graefelfing, Germany). The signal was detected using an Andor iXonEM+ 897 (back-illuminated) EMCCD camera (16 μm pixel size). The following filter sets were used: dichroic filter (ZT405/488/561/640rpc, Chroma, Olching, Germany), emission filter (446/523/600/677 nm BrightLine quad-band band-pass filter, Semrock, Rochester, NY, USA), and an additional emission filter (HQ 700/75 M, NC209774, Chroma Technology GmbH, Olching, Germany). Simultaneous two-color imaging was carried out using a dual emission filter cube (OptoSplit II image splitter, Cairn Research, 525/50, 675/50). The 3D localization of EVs was analyzed using a custom-built software: 3D STORM tools.^49^

### Stepwise Photobleaching

eGFP-CD63 EVs were excited using a diode laser (488 nm, Toptica Photonics, Graefelfing, Germany). In addition to the above-described optical path, the fluorescence signal was filtered with an emission filter (525 ± 25 nm, Semrock, US). A sequence of 500 images with an illumination time of 20 ms at a laser intensity of 5 kW/cm^2^ was acquired. The signals of individual EVs were analyzed using a previously described single-molecule analysis platform.^50^ A detailed description is available in the Supporting Information.

### Cellular EV Uptake

HeLa cells were cultured in Dulbecco’s Modified Eeagle’s Medium (DMEM, Thermo Fisher, Waltham, MA, USA), high glucose, supplemented with 10% fetal bovine serum (FBS; Thermo Fisher) and 1% penicillin/streptomycin (complete growth medium) at 37 °C in a 5% CO_2_ atmosphere. HeLa cells were seeded in Lab-Tek 8-well chambered cover glasses (Thermo Fisher, Waltham, USA) or on self-prepared chambers pasted on glass slides with 2-component glue. The glass slides were coated with 5 μg/cm^2^ poly-d-lysine (PDL; Advanced BioMatrix, Carlsbad, CA, USA) or 1 μg/cm^2^ fibronectin (Sigma-Aldrich, St. Louis, MO, USA). Cells were cultured in complete growth medium for 48 h (80–90% confluency). Cells were washed once 2 h before the experiment, and the medium was changed to low-fluorescence FluoroBrite medium (Gibco, Carlsbad, CA, USA) supplemented with 1% EV-depleted FBS to reduce autofluorescence and starve the cells. eGFP-CD63 EVs were diluted 1:100 in PBS, added to the cells, and incubated for 30–60 min at 37 °C in 5% CO_2_ atmosphere. To label the outer cell membrane, anti-CD59 monoclonal antibody conjugated with AlexaFluor 647 (Thermo Fisher, Waltham, MA, USA) was added for 10 min and washed prior to imaging.

### 3D Tracking

Tracking of EVs was performed using a modified version of Trackpy software.^51^ A full description of the Trackpy tool is available at http://soft-matter.github.io/trackpy/v0.5.0/. In this study, single-molecule localization data (obtained by 3D STORM tools) was used as an input for Trackpy. The following parameters in the Trackpy software were applied: maximum distance that the fluorescence signals can move between frames (i.e., search range) was 480 nm; minimum trajectory length was set to zero; maximum number of frames that a fluorescence signal can disappear and still be considered the same track was set to 0 (tracks with missing frames were discarded).

### Mean Square Displacement (MSD) Analysis

Diffusion analysis was performed by a custom written MATLAB platform (based on @msdanalyzer).^52^ The spreadsheets with linked coordinates (output from Trackpy tool) were used as input for @msdanalyzer. For the colocalization analysis of internalized EVs and transferrin from human serum conjugated with AlexaFluor647 (Invitrogen, Carlsbad, CA, USA), HeLa cells were cultured for 48 h on glass slides coated with 1 μg/cm^2^ fibronectin. eGFP-CD63 EVs were diluted 1:100 in FluoroBrite medium supplemented with 1% EV-depleted FBS and incubated with HeLa cells for 30 min at 37 °C in 5% CO_2_ atmosphere. After EVs were internalized by the cells, transferrin was added to the cells and incubated for 10 min of incubation at 37 °C, 5% CO_2_. Cells were washed and fixed with 4% paraformaldehyde (PFA) in PBS. Imaging was performed in OxEA buffer (50 mM β-mercaptoethylamine, 3% oxyrase, 100 μM DL-lactate, 30v/v% glycerine) in PBS adjusted to pH 8.0–8.5 with NaOH. Images were acquired as previously described. Samples were illuminated for 20 ms, and a sequence of 10 000 frames was recorded with a delay between individual images of 33 ms. During the camera chip read-out, UV light (405 nm diode laser, Toptica Photonics) was additionally used to activate the probes, and the laser power was adjusted during the measurements accordingly.

### AFM-FM Colocalization Analysis

An AFM instrument (JPK NanoWizard 4) mounted on an inverted optical microscope (Zeiss Axio Observer) was used for sample characterization. JPK QI mode was used to record complete force–distance curves in each measured pixel. In all measurements, MLCT-F cantilevers (Bruker) with a nominal tip radius of 20 nm and a spring constant of 0.6 N/m were used. The indentation force was set to 0.8 nN. Height and Young’s modulus (elasticity) were extracted from force–distance curves via JPK data processing software. Young’s modulus was obtained by fitting Hertz-contact model (paraboloid tip shape; tip radius of 20 nm; Poisson’s ratio of 0.45). All measurements were conducted in PBS at room temperature.

**Figure 1:**
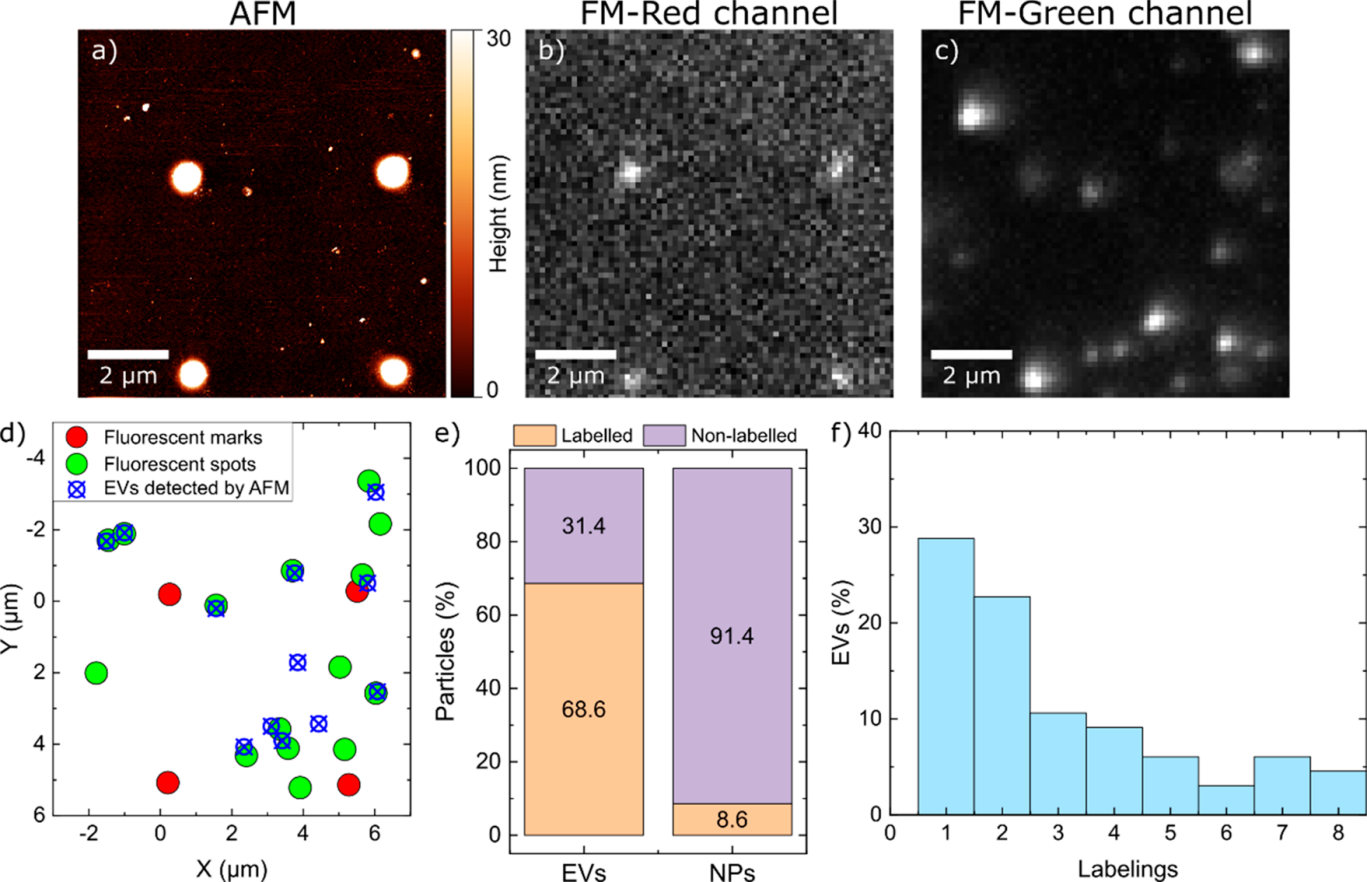
Characterization of EVs. (a) AFM topographic image of the purified EV sample adsorbed to a glass coverslip with polymerized grid marks for orientation. (b and c) Fluorescence images of the same area presented in panel a, red and green channels, respectively. (d) Colocalization map of EVs detected via AFM in panel a and fluorescent emitting events of panels b and c. (e) Labeling ratio of EVs (*n* = 86) and other NPs (*n* = 350) detected within a sample area of ∼500 μm^2^. (f) Histograms displaying the number of eGFPs per single EV analyzed on a glass substrate, determined via SP (500 images, illumination time = 20 ms, *I* = 5 kW/cm^2^).

As an excitation source for fluorescence measurements, a 491 nm diode laser (Cobolt Calypso 100) was used. A 525 ± 25 nm bandpass filter (for detection of eGFP) or 700 ± 25 nm bandpass filter (for detection of marks) was inserted in the detection path. Alignment marks were polymeric dots structured via multiphoton lithography with high autofluorescence.^53^ Alignment of the AFM and FM images was done in several steps: (1) Coordinates of all particles and marks were extracted from AFM images by Gwyddion software (v2.58).^54^ (2) Coordinates of all fluorescent events (including the marks) were extracted from FM images by 3D STORM tool software.^48^ (3) Transformation (shift, rotation, and scaling) was applied to the FM image of the fluorescence marks coordinates until it fit the marks on the AFM image. (4) The same transformation was applied to the FM image of the EVs. (5) The maps of AFM and FM detected position were plotted on one graph as presented in Figure 1d.

### 3D Single-Molecule Two-Color Colocalization Analysis

Colocalization analysis was performed by a custom written MATLAB platform available at https://github.com/CURTLab. Single-molecule localization data (obtained by 3D STORM tools) was used as an input. Fiducial fluorescent marker signals (tetraspeck beads) measured in two-color channels were used as a reference. The detailed description of the MATLAB routine is presented in the Supporting Information.

## Results and Discussion

### Quantitative Analysis of EVs by Combining AFM and SMFM

We used a combination of AFM and SMFM to analyze an isolated EV population with regard to their size and labeling ratio. We isolated EVs by sequential ultracentrifugation, which classifies them as the small EV-fraction according to the Minimal Information for Studies of Extracellular Vesicles (MISEV).^55^ For simplicity, we will use the general term “EVs” for our sample. Sparsely distributed single EVs were immobilized on glass coverslips. The quantitative imaging (QI) mode of our AFM allowed us to simultaneously capture the topography (Figure 1a) and the Young’s modulus (Figure SI 1b) of the samples. Acquiring both parameters is crucial for the distinction of EVs from other nanoparticles (NPs) (by the height and/or Young’s modulus distribution).^41^Figure SI 1c shows the height distribution of 436 particles. AFM data enabled a clear separation of EVs from surrounding NPs (e.g., cell debris and damaged EVs). The mean size for the EVs and NPs was 45.5 ± 14.5 and 11.8 ± 5.5 nm, respectively. Application of SMLM allowed for determination of the eGFP-emitter positions on the glass slide (Figure 1c) where the simultaneously obtained SMFM and AFM images were aligned using a multiphoton lithography^56^ structured grid of fluorescent fiducials (Figure 1a,b and Figure SI 1a; more details are provided in Materials and Methods). By correlating the EVs’ positions extracted from AFM data with the positions of the fluorophores extracted from the SMFM data (Figure 1d), we were able to distinguish between labeled and unlabeled EVs as well as between labeled and unlabeled NPs. Figure 1e shows the percentage of labeled EVs and other NPs measured within a representative area of ∼500 μm^2^. Nearly 68% of all identified EVs were fluorescently labeled, while in contrast only 8% of NPs showed a fluorescence signal. The numbers of detected EVs and NPs in the sample were *n*_EVs_ = 86 and *n*_NPs_ = 350, respectively. Information about labeling efficiency of EVs (number of CD63-eGFP positive EVs) is necessary to correctly estimate uptake efficiency/pathways. Moreover, the presence of labeled contaminants in the EV solution (NPs) should also be taken into account. The contribution of labeled NPs (possibly eGFP-CD63 bound to cell debris or pure eGFP) regarding their cell uptake is difficult to predict. However, we have shown that purified eGFP is taken up ∼10× times less efficiently compared to the EVs.^44^

We further determined the total number of eGFPs per EV by counting the photobleaching steps in a stepwise photobleaching (SP) experiment.^57−62^ Panels a and b of Figure SI 2 depict examples of SP measured on a glass substrate and inside the cells, respectively. One of SP’s main advantages is its independence from environmental conditions, which in contrast to other methods^44^ makes it highly suited for intracellular studies. To estimate the total number of labeled transmembrane CD63 proteins per EV, asequences of 500 images with an illumination time of 20 ms were acquired. The average intensity of a single GFP-CD63 EV on the glass substrate was ∼700 ± 150 counts/pixel. Figure SI 2c represents one of the recorded raw fluorescence images that display randomly distributed, immobilized EVs on the glass substrate. The photobleaching steps of individual EVs were analyzed within the sequences using our analysis platform, Spotty^50^ (additional information is in the Supporting Information). The resulting average number of eGFPs per EV was 2.9 ± 0.25 (standard error of mean), and the SNR was 22 ± 9. The resulting histogram of eGFPs per EV is shown in Figure 1f: 28.8% of the total detected fluorescence signal corresponded to a single eGFP signal and thus single CD63 proteins,^44^ and 22.7% showed two eGFP labels per EV; the remaining population of 48.5% represents fluorescence signals corresponding to three or more eGFPs per EV.

### Uptake of EVs by HeLa Cells

**Figure 2:**
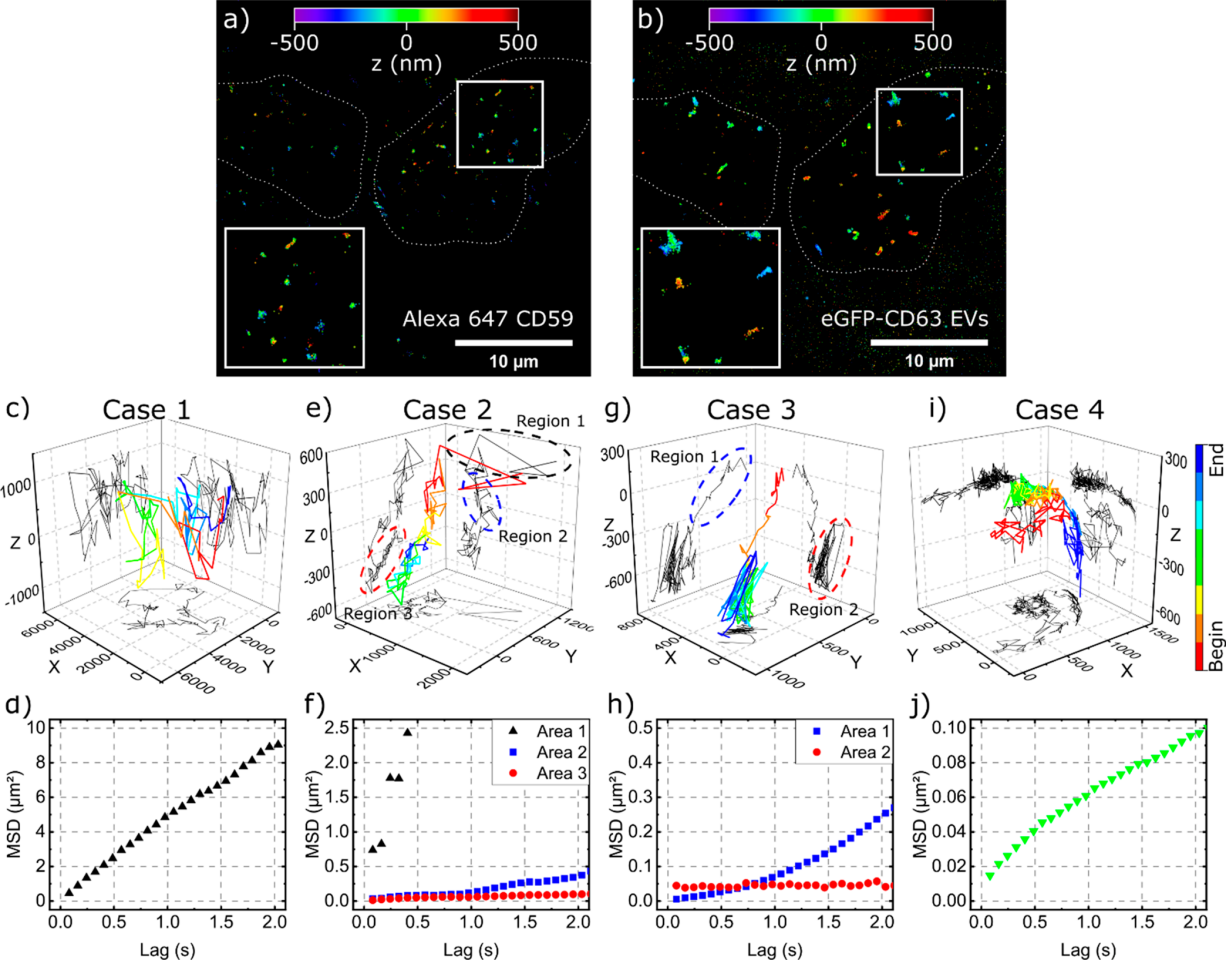
3D localization and diffusion dynamics of EVs in HeLa cells. (a) 3D localization of CD59 on the cell membrane with PA_*xy*_= 38.3 nm and PA_*z*_= 99.4 nm. (b) 3D localization of EVs with PA_*xy*_= 32.7 nm and PA_*z*_ = 56.7 nm. White dotted lines show the cell outline and a zoom of the localized eGFP-CD63 EVs and CD59 position is presented in the insets of both images. (c) A trajectory of freely diffusing EV in the solution above or near the cells (case 1). The plot shows 3D trajectory (color coded direction of movement) as well as its *xy*, *yz*, and *xz* projections. (d) MSD plots for the trajectory shown in panel c. (e and f) Trajectory and MSD plots of an EV approaching cell membrane, passing through it, and subsequently diffusing inside the cell (case 2). Different motion regions are indicated with dashed ellipses. (g and h) Trajectory and MSD plots of an EV entering the cell and localized somewhere inside the cell (case 3). (i and j) Trajectory and MSD plots of an EV diffusing along the cell membrane without entering the cell. For all trajectories, the line color indicates the direction of the EV movement during acquisition (starting point is in red and the end in blue). The *xy*, *yz*, and *xz* projections are shown in black.

For studying cellular uptake of EVs in HeLa cells, it is essential to clearly distinguish between the extra- and intracellular spaces. Toward this end, we labeled the GPI anchored CD59 glycoprotein with an Alexa 647 conjugated anti-CD59 antibody to determine the *z*-position of the cell membrane. The fluorescence signals of the Alexa 647 conjugated anti-CD59 antibodies diffusing in the membrane were observed over time, and the membrane’s curvature was extrapolated. Figure 2a shows a pair of cells with localized positions of the tracked CD59 molecules (*n* = 16 ± 10 per cell; position accuracy (PA) of 38 nm in the *xy*-direction and 99 nm in the *z*-direction). The movement of the cell membrane in the axial position was monitored over the acquisition time and typically was in the range of PA_*z*_ (∼100 nm, Figure SI 3). After the membrane stain, cells were incubated with EVs, and 3D trajectories were obtained upon localization of the emitter positions at a subdiffraction level. The same cells with tracked and localized EVs (*n* = 25 ± 14 per cell, lateral PA_*xy*_ of 32 nm and axial PA_*z*_ of 56 nm) are shown in Figure 2b. Consequently, we were able to distinguish between the EVs approaching the cell from extracellular regions and their transport across the membrane. The 3D tracking information was used for a detailed study of the EVs’ diffusion (Materials and Methods). The diffusion behavior of the eGFP-CD63 EVs has been quantified for each trajectory. In general, the diffusion trajectories are divided into two main groups: rather fast and rather slow tracks, with the ratio (fast:slow) around 1:1.5 (in total ∼700 tracks were considered). We have assigned fast tracks to the EVs outside the cells and slow track to EVs inside or in the vicinity of the cells. However, there are also mixed tracks (starting fast and slowing down closer the end), which most probably represent the uptake of the EVs by cells (crossing membrane border).

Figure 2c–j shows a representative exemplary trajectory of most of these cases. We distinguished between four cases: Case 1: The EV freely diffuses in the solution outside the cells (Figure 2c). This is represented by large movements in all three directions. The mean square displacement (MSD) analysis (Figure 2d) shows a Brownian motion with diffusion coefficient *D* = 0.729 ± 0.008 μm^2^/s. The observed diffusion coefficients are smaller compared to the theoretical values (5 μm^2^/s, by the Stokes–Einstein relation, *D* = *kBT*(6πη*r*)^−1^) as well as experimental values (3 μm^2^/s on average) reported earlier,^63^ but they are larger compared to the movement of molecules bound to the plasma membrane (0.002–0.1 μm^2^/s).^64−66^ The observed three-dimensional movement behavior (Figure 2c) excludes the possibility of membrane attachment; however, the dynamics of the EV outside of the cell might be influenced by the extracellular matrix (e.g. glycocalyx) as well as cell culture medium viscosity.^67^ Case 2 (Figure 2e): An EV which approached the cell membrane from the extracellular space (region 1), diffused across the membrane (region 2), and subsequently diffused inside the cell (region 3). For the MSD calculation, the trajectory was split manually into the corresponding three subtrajectories (indicated in the 3D plot by dotted ellipses) and analyzed separately (Figure 2f). The MSD curve of region 1 suggests Brownian diffusion with diffusion coefficient *D* = 0.89 ± 0.16 μm^2^/s, which is similar to the value detected for particles outside the cells. In contrast, region 2 shows directed motion with a characteristic speed *v* = 0.229 ± 0.013 μm/s. The region 3 (inside the cell) diffusion behavior changed back to Brownian motion (*D* = 0.0063 ± 0.0003 μm^2^/s) with slight asymptotic behavior, indicating confinement of the EV inside the cell. Case 3: The EV passed across the cell membrane and moved inside the cell where it finally was localized (Figure 2g). In this case, two different motions could be observed. After crossing the cell membrane, EVs change the motion from directed to Brownian, which is visible in the MSD analysis (Figure 2h). The quadratic time dependence of the first region yielded a speed of *v* = 0.212 ± 0.002 μm/s, which is similar to the previous case and in accordance with other studies, where vesicle transport along microtubules have been observed.^68^ The constant value of MSD at the second part (region 2) of the trajectory indicates immobilization of EVs inside the cell. Case 4: An EV is diffusing along the cell membrane without entering the cell. Although the measured track was relatively long (∼35 s), the observed EV did not enter the cell. Possible reasons could be the tracked particle was not an EV but a fluorophore alone or a damaged EV. Therefore, no active uptake pathway can be observed, or our measurement time window was smaller than time required for unspecific uptake. Second, the tracked EV was intact but lacked the necessary binding protein for active uptake.

### 3D Nanoscale Colocalization of EVs and Transferrin Inside HeLa Cells

**Figure 3:**
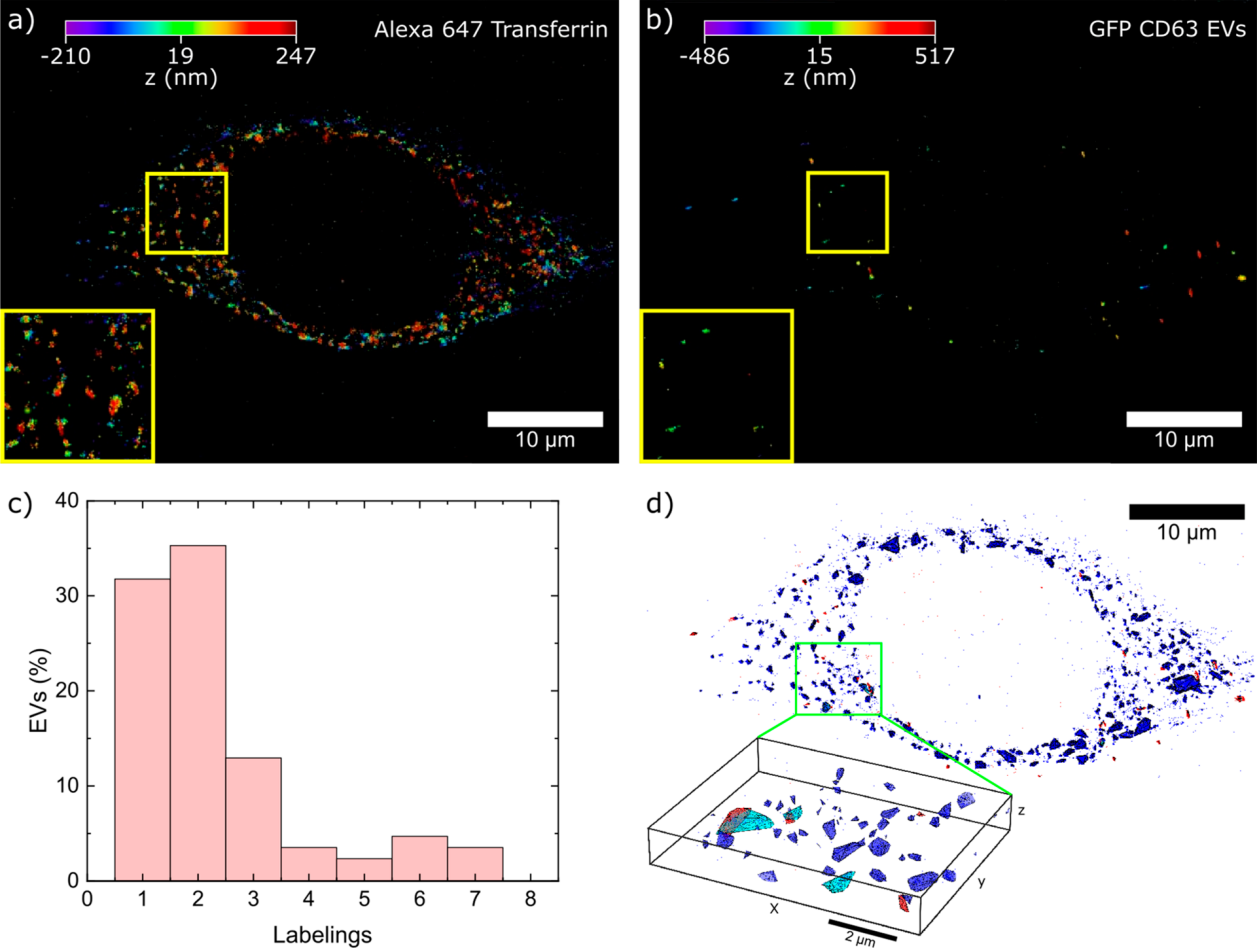
Colocalization of EVs with respect to AF647–transferrin in HeLa cells. (a) dSTORM image of AF647–transferrin localized in HeLa cells with a lateral PA_*xy*_ of 37.1 nm and axial PA_*z*_ of 45.6 nm. (b) dSTORM image of eGFP-CD63 EVs internalized in HeLa cells localized with a lateral PA_*xy*_ of 38.2 nm and axial PA_*z*_ of 54.5 nm. Insets show a magnified area of the localized images (marked by yellow squares in panels a and b). 10 000 frames were recorded in two-colors simultaneously (*t*_ill_ = 20 ms, *I* = 4.2 kW/cm^2^ and 7 kW/cm^2^ for EVs and AF647–transferrin imaging, respectively). (c) Histograms of eGFPs signals originating from EVs inside HeLa cells (internalized after incubation for ∼30 min). Data were acquired by SP (500 images, until = 20 ms, *I* = 5 kW/cm^2^). (d) Colocalization image of AF647–transferrin (blue) and eGFP-CD63 EVs (red) after correcting for chromatic aberrations. Inset shows a magnified area of the colocalized image (marked with green box) where light blue labeled AF647–transferrin clusters overlapped with EVs.

To demonstrate the capabilities of our experimental instrument to resolve potential EV uptake mechanisms, we performed 3D colocalization experiments of eGFP-CD63 EVs and fluorescently labeled transferrin with Alexa 647 (AF647–transferrin), a marker for the early/recycling endosomal compartment. Transferrin binds to the transferrin receptor on the cell surface, and the complex enters the cell through clathrin-coated pit-mediated endocytosis and is further directed to early endosomes.^69,70^ The 3D nanoscopic colocalization was performed for 3D dSTORM two-color localization data with average PA_*xy*_ and PA_*z*_ of 38 and 54 nm for eGFP-CD63 EVs and 37 and 45 nm for AF647–transferrin, respectively. HeLa cells were incubated with EVs for ∼30 min at 37 °C, and after EV internalization, cells were fixed and incubated with AF647–transferrin for 10 min (to reduce unspecific binding) for imaging. The sparsely distributed eGFP-CD63 EV signals inside HeLa cells were then localized. Figure 3a,b shows corresponding dSTORM images of AF647–transferrin and eGFP-CD63 EVs. To determine whether internalized EVs aggregate inside HeLa cells, we quantified the corresponding distribution in SP experiments and compared it to the distribution determined for EVs on a glass coverslip (Figure 1f). Figure SI 2d depicts a fluorescence image of fixed HeLa cells, incubated with eGFP-CD63 EVs. The fluorescence signal of a single eGFP-CD63 EV inside the cell was 600 ± 330 counts/pixel (*t*_ill_ = 20 ms, *I* = 4.2 kW/cm^2^) with an SNR of 18 ± 4. The histogram determined from the SP analysis (Figure 3c) represents the total number of eGFPs per detected fluorescent signal inside the cells (*N*_cells_ = 10), indicating on average 2.4 ± 0.2 (standard error of mean) eGFPs per EV. The SP analysis revealed that 31.8% of the detected fluorescence signals correspond to one eGFP molecule, similar to what has been observed for eGFP-CD63 EVs on a glass substrate. 35.3% of the fluorescence signals detected in cells had twice the eGFP intensity, a value 10% larger compared to signals derived from eGFP-CD63 EVs on a glass substrate. The remaining 32.9% of the fluorescent signals in cells correspond to three or more eGFP molecules within a diffraction-limited volume. Overall, the intensity distribution of internalized eGFP-CD63 EVs was not significantly different from sparsely distributed eGFP-CD63 EVs on a glass substrate. Thus, we assume that the signals obtained in cells correspond to taken up eGFP-CD63 EVs and not to its aggregates. To analyze the colocalization of eGFP-CD63 EVs with AF647–transferrin, we developed an image processing software for two-color colocalization analysis (see the Supporting Information). The software is based on individual cluster analysis of localized emitter positions. Each cluster in the EV image is tested for intersections or overlap with the clusters in the AF647–transferrin image. The test is performed by computing the convex hull intersection of the EV clusters with K convex hulls constructed for its closest neighbors among the cluster of AF647–transferrin localization positions. The closest neighbors of the tested EV cluster are determined based on the distance between its centroid and the centroids of the clusters from the AF647–transferrin. Figure 3c,d shows the colocalization images of eGFP-CD63 EVs with AF647–transferrin as well as a magnification thereof (green box in panel c). In the magnified image, the clusters of the AF647–transferrin which colocalize with EVs are represented in light blue. For the overlap analysis, localization precision of single emitters has been considered (see the Supporting Information). The analysis yields that 25.3% of all EVs show colocalization (*n* = 185 localized EVs in several cells) with AF647–transferrin.

## Conclusion

The quantitative analysis pipeline presented in this work combines data obtained from AFM and 2D SMFM as well as 3D SMLM. This yields a precise quantification of the purified, fluorescently labeled, endosome-derived EVs which were later incubated with HeLa cells. First, AFM and SMFM were used to image simultaneously the same area, providing information on the localization and intensity of the individual emitters. Thus, we were able to differentiate between EVs and other NPs based on their fluorescence signal and topography. A fraction of 68% carried a label; the rest were either unlabeled or non-EVs. Of the labeled fraction, 29% were carrying a single eGFP (the rest had multiple labels), indicating the presence of only one labeled CD63 in the membrane.

In a showcase study, EVs were incubated with HeLa cells, and their uptake was analyzed in three dimensions. 3D two-color nanoscopy was key to gain a full picture of particle diffusion, especially dynamic capturing of the eGFP-CD63 EV uptake events across the cell membrane as well as colocalization of the eGFP-CD63 EVs with other organelles. 3D colocalization of two-color SMLM data supported by a newly developed colocalization software allowed quantitative studies of GFP-CD63 EVs and transferrin up-take. The analysis yielded a 25% 3D overlap of eGFP-CD63 EVs with transferrin. Additionally, SP data inside the cells showed no aggregation of the taken up eGFP-CD63 EVs (compared to the preanalyzed eGFP-CD63 EV population). Combined colocalization and SP analysis gives information about delivery of the eGFP-CD63 EVs (or EV components) to the organelles in individual cells. This information is key for future work addressing uptake pathway analysis and will provide information about targeted delivery of eGFP-CD63 EVs. Nevertheless, it has to be taken into consideration that 68% of all eGFP-CD63 EVs carried a fluorescent label. The remeining 32% of unlabeled EVs can not be observed but potentially can increase the colocalization results. Additionally, it can be assumed that the CD63 N-terminally fused with eGFP does not influence the protein function and thus a possible uptake pathway.

In addition, all the experiments were carried out with the same EV sample obtained from a single purification run. This was possible due to at least a 100-fold or lower sample amount needed (<10^9^ EVs/ml) here compared to each of the most standard EV quantification methods (e.g., nanoparticle tracking analysis or Western blot) described in MISEV.^55^

In summary, quantification of aggregation, uptake dynamics, and colocalization at a single-molecule level of the taken-up EVs with cellular compartments and proteins provide added value which is essential to deciphering biological processes related to EVs at the single-cell level. The presented toolbox allows considering the intrinsic heterogeneity of the EV population by its precise precharacterization. It can address numerous biotechnological and biological questions, such as EV population-dependent uptake mechanisms, the impact of storage on EV uptake, difference in diffusion dynamics of different EVs, or delivery of EVs to cellular compartments. We hope that the presented experimental pipeline will inspire and initiate further studies in the EV community.

## Supporting Information Available

Additional information about AFM/FM image colocalization, a detailed explanation of the stepwise photobleaching experiment and analysis, simulation of the of two-color correction, and a detailed description of the 3D single-molecule two-color colocalization analysis (PDF)

## References

[ref1] Kalluri R.; LeBleu V. S. The Biology, Function, and Biomedical Applications of Exosomes. Science 2020, 367 (6478), eaau6977 10.1126/science.aau6977. 32029601 PMC7717626

[ref2] Goreham R. V.; Ayed Z.; Ayupova D.; Dobhal G. Extracellular Vesicles: Nature’s Own Nanoparticles. Comprehensive Nanoscience and Nanotechnology 2019, 1–5, 27–48. 10.1016/B978-0-12-803581-8.10412-6.

[ref3] Yáñez-Mó M.; Siljander P. R. M.; Andreu Z.; Zavec A. B.; Borràs F. E.; Buzas E. I.; Buzas K.; Casal E.; Cappello F.; Carvalho J.; Colás E.; Cordeiro-Da Silva A.; Fais S.; Falcon-Perez J. M.; Ghobrial I. M.; Giebel B.; Gimona M.; Graner M.; Gursel I.; Gursel M.; Heegaard N. H. H.; Hendrix A.; Kierulf P.; Kokubun K.; Kosanovic M.; Kralj-Iglic V.; Krämer-Albers E. M.; Laitinen S.; Lässer C.; Lener T.; Ligeti E.; Line A.; Lipps G.; Llorente A.; Lötvall J.; Manček-Keber M.; Marcilla A.; Mittelbrunn M.; Nazarenko I.; Nolte-’t Hoen E. N. M.; Nyman T. A.; O’Driscoll L.; Olivan M.; Oliveira C.; Pállinger É.; Del Portillo H. A.; Reventós J.; Rigau M.; Rohde E.; Sammar M.; Sánchez-Madrid F.; Santarém N.; Schallmoser K.; Ostenfeld M. S.; Stoorvogel W.; Stukelj R.; Van Der Grein S. G.; Helena Vasconcelos M.; Wauben M. H. M.; De Wever O. Biological Properties of Extracellular Vesicles and Their Physiological Functions. Journal of Extracellular Vesicles 2015, 4 (2015), 27066 10.3402/jev.v4.27066. 25979354 PMC4433489

[ref4] Serrano-Pertierra E.; Blanco-López M. C. Extracellular Vesicles: From Biology to Biomedical Applications. Bioengineering 2019, 6 (3), 79 10.3390/bioengineering6030079. 31489953 PMC6783948

[ref5] Van Niel G.; D’Angelo G.; Raposo G. Shedding Light on the Cell Biology of Extracellular Vesicles. Nat. Rev. Mol. Cell Biol. 2018, 19 (4), 213–228. 10.1038/nrm.2017.125. 29339798

[ref6] Valadi H.; Ekström K.; Bossios A.; Sjöstrand M.; Lee J. J.; Lötvall J. O. Exosome-Mediated Transfer of MRNAs and MicroRNAs Is a Novel Mechanism of Genetic Exchange between Cells. Nat. Cell Biol. 2007, 9 (6), 654–659. 10.1038/ncb1596. 17486113

[ref7] Tetta C.; Ghigo E.; Silengo L.; Deregibus M. C.; Camussi G. Extracellular Vesicles as an Emerging Mechanism of Cell-to-Cell Communication. Endocrine 2013, 44 (1), 11–19. 10.1007/s12020-012-9839-0. 23203002 PMC3726927

[ref8] Théry C. Cancer: Diagnosis by Extracellular Vesicles. Nature 2015, 523 (7559), 161–162. 10.1038/nature14626. 26106856

[ref9] Revenfeld A. L. S.; Bæk R.; Nielsen M. H.; Stensballe A.; Varming K.; Jo̷rgensen M. Diagnostic and Prognostic Potential of Extracellular Vesicles in Peripheral Blood. Clinical Therapeutics 2014, 36 (6), 830–846. 10.1016/j.clinthera.2014.05.008. 24952934

[ref10] De Jong O. G.; Kooijmans S. A. A.; Murphy D. E.; Jiang L.; Evers M. J. W.; Sluijter J. P. G.; Vader P.; Schiffelers R. M. Drug Delivery with Extracellular Vesicles: From Imagination to Innovation. Acc. Chem. Res. 2019, 52 (7), 1761–1770. 10.1021/acs.accounts.9b00109. 31181910 PMC6639984

[ref11] Herrmann I. K.; Wood M. J. A.; Fuhrmann G. Extracellular Vesicles as a Next-Generation Drug Delivery Platform. Nat. Nanotechnol. 2021, 16 (7), 748–759. 10.1038/s41565-021-00931-2. 34211166

[ref12] Elsharkasy O. M.; Nordin J. Z.; Hagey D. W.; de Jong O. G.; Schiffelers R. M.; Andaloussi S. EL; Vader P. Extracellular Vesicles as Drug Delivery Systems: Why and How?. Adv. Drug Delivery Rev. 2020, 159, 332–343. 10.1016/j.addr.2020.04.004. 32305351

[ref13] Vader P.; Mol E. A.; Pasterkamp G.; Schiffelers R. M. Extracellular Vesicles for Drug Delivery. Adv. Drug Delivery Rev. 2016, 106, 148–156. 10.1016/j.addr.2016.02.006. 26928656

[ref14] de Jong O. G.; van Balkom B. W. M.; Schiffelers R. M.; Bouten C. V. C.; Verhaar M. C. Extracellular Vesicles: Potential Roles in Regenerative Medicine. Frontiers in Immunology 2014, 5 (DEC), 608 10.3389/fimmu.2014.00608. 25520717 PMC4253973

[ref15] Casado-Díaz A.; Quesada-Gómez J. M.; Dorado G. Extracellular Vesicles Derived From Mesenchymal Stem Cells (MSC) in Regenerative Medicine: Applications in Skin Wound Healing. Frontiers in Bioengineering and Biotechnology 2020, 8 (March), 146 10.3389/fbioe.2020.00146. 32195233 PMC7062641

[ref16] Cocucci E.; Meldolesi J. Ectosomes and Exosomes: Shedding the Confusion between Extracellular Vesicles. Trends in Cell Biology 2015, 25 (6), 364–372. 10.1016/j.tcb.2015.01.004. 25683921

[ref17] Colombo F.; Norton E. G.; Cocucci E. Microscopy Approaches to Study Extracellular Vesicles. Biochimica et Biophysica Acta - General Subjects 2021, 1865 (4), 129752 10.1016/j.bbagen.2020.129752. 32991970

[ref18] Andreu Z.; Yáñez-Mó M. Tetraspanins in Extracellular Vesicle Formation and Function. Frontiers in Immunology 2014, 5 (SEP), 442 10.3389/fimmu.2014.00442. 25278937 PMC4165315

[ref19] Jankovičová J.; Sečová P.; Michalková K.; Antalíková J. Tetraspanins, More than Markers of Extracellular Vesicles in Reproduction. International Journal of Molecular Sciences 2020, 21 (20), 7568 10.3390/ijms21207568. 33066349 PMC7589920

[ref20] Mulcahy L. A.; Pink R. C.; Carter D. R. F. Routes and Mechanisms of Extracellular Vesicle Uptake. Journal of Extracellular Vesicles 2014, 3 (1), 24641 10.3402/jev.v3.24641. PMC4122821 25143819

[ref21] Kwok Z. H.; Wang C.; Jin Y. Extracellular Vesicle Transportation and Uptake by Recipient Cells: A Critical Process to Regulate Human Diseases. Processes 2021, 9 (2), 273 10.3390/pr9020273. 34336602 PMC8323758

[ref22] Bonsergent E.; Grisard E.; Buchrieser J.; Schwartz O.; Théry C.; Lavieu G. Quantitative Characterization of Extracellular Vesicle Uptake and Content Delivery within Mammalian Cells. Nat. Commun. 2021, 12 (1), 1864 10.3390/pr9020273. 33767144 PMC7994380

[ref23] Liu Z.; Lavis L. D.; Betzig E. Imaging Live-Cell Dynamics and Structure at the Single-Molecule Level. Mol. Cell 2015, 58 (4), 644–659. 10.1016/j.molcel.2015.02.033. 26000849

[ref24] Panagopoulou M. S.; Wark A. W.; Birch D. J. S.; Gregory C. D. Phenotypic Analysis of Extracellular Vesicles: A Review on the Applications of Fluorescence. Journal of Extracellular Vesicles 2020, 9 (1), 1710020 10.1080/20013078.2019.1710020. 32002172 PMC6968689

[ref25] Jiang C.; Yang M.; Li W.; Dou S.-X.; Wang P.-Y.; Li H. Spatiotemporal Three-Dimensional Transport Dynamics of Endocytic Cargos and Their Physical Regulations in Cells. iScience 2022, 25 (5), 104210 10.1016/j.isci.2022.104210. 35479412 PMC9035719

[ref26] Low-Nam S. T.; Lidke K. A.; Cutler P. J.; Roovers R. C.; van Bergen en Henegouwen P. M. P.; Wilson B. S.; Lidke D. S. ErbB1 Dimerization Is Promoted by Domain Co-Confinement and Stabilized by Ligand Binding. Nat. Struct Mol. Biol. 2011, 18 (11), 1244–1249. 10.1038/nsmb.2135. 22020299 PMC3210321

[ref27] Heusermann W.; Hean J.; Trojer D.; Steib E.; von Bueren S.; Graff-Meyer A.; Genoud C.; Martin K.; Pizzato N.; Voshol J.; Morrissey D. V.; Andaloussi S. E. L.; Wood M. J.; Meisner-Kober N. C. Exosomes Surf. on Filopodia to Enter Cells at Endocytic Hot Spots, Traffic within Endosomes, and Are Targeted to the ER. J. Cell Biol. 2016, 213 (2), 173–184. 10.1083/jcb.201506084. 27114500 PMC5084269

[ref28] Chuo S. T. Y.; Chien J. C. Y.; Lai C. P. K. Imaging Extracellular Vesicles: Current and Emerging Methods. Journal of Biomedical Science 2018, 25 (1), 91 10.1186/s12929-018-0494-5. 30580764 PMC6304785

[ref29] Corso G.; Heusermann W.; Trojer D.; Görgens A.; Steib E.; Voshol J.; Graff A.; Genoud C.; Lee Y.; Hean J.; Nordin J. Z.; Wiklander O. P. B.; El Andaloussi S.; Meisner-Kober N. Systematic Characterization of Extracellular Vesicle Sorting Domains and Quantification at the Single Molecule – Single Vesicle Level by Fluorescence Correlation Spectroscopy and Single Particle Imaging. Journal of Extracellular Vesicles 2019, 8 (1), 1663043 10.1080/20013078.2019.1663043. 31579435 PMC6758720

[ref30] Malenica M.; Vukomanović M.; Kurtjak M.; Masciotti V.; Dal Zilio S.; Greco S.; Lazzarino M.; Krušić V.; Perčić M.; Badovinac I. J.; Wechtersbach K.; Vidović I.; Baričević V.; Valić S.; Lučin P.; Kojc N.; Grabušić K. Perspectives of Microscopy Methods for Morphology Characterisation of Extracellular Vesicles from Human Biofluids. Biomedicines 2021, 9 (6), 603 10.3390/biomedicines9060603. 34073297 PMC8228884

[ref31] Saari H.; Lisitsyna E.; Rautaniemi K.; Rojalin T.; Niemi L.; Nivaro O.; Laaksonen T.; Yliperttula M.; Vuorimaa-Laukkanen E. FLIM Reveals Alternative EV-Mediated Cellular up-Take Pathways of Paclitaxel. J. Controlled Release 2018, 284, 133–143. 10.1016/j.jconrel.2018.06.015. 29906554

[ref32] Pomatto M. A. C.; Bussolati B.; D’Antico S.; Ghiotto S.; Tetta C.; Brizzi M. F.; Camussi G. Improved Loading of Plasma-Derived Extracellular Vesicles to Encapsulate Antitumor MiRNAs. Molecular Therapy - Methods & Clinical Development 2019, 13, 133–144. 10.1016/j.omtm.2019.01.001. 30788382 PMC6370572

[ref33] Panagopoulou M. S.; Wark A. W.; Birch D. J. S.; Gregory C. D. Phenotypic Analysis of Extracellular Vesicles: A Review on the Applications of Fluorescence. Journal of Extracellular Vesicles 2020, 9 (1), 1710020 10.1080/20013078.2019.1710020. 32002172 PMC6968689

[ref34] Nizamudeen Z.; Markus R.; Lodge R.; Parmenter C.; Platt M.; Chakrabarti L.; Sottile V. Rapid and Accurate Analysis of Stem Cell-Derived Extracellular Vesicles with Super Resolution Microscopy and Live Imaging. Biochimica et Biophysica Acta - Molecular Cell Research 2018, 1865 (12), 1891–1900. 10.1016/j.bbamcr.2018.09.008. 30290236 PMC6203808

[ref35] Lennon K. M.; Wakefield D. L.; Maddox A. L.; Brehove M. S.; Willner A. N.; Garcia-Mansfield K.; Meechoovet B.; Reiman R.; Hutchins E.; Miller M. M.; Goel A.; Pirrotte P.; Van Keuren-Jensen K.; Jovanovic-Talisman T. Single Molecule Characterization of Individual Extracellular Vesicles from Pancreatic Cancer. Journal of Extracellular Vesicles 2019, 8 (1), 1685634 10.1080/20013078.2019.1685634. 31741725 PMC6844376

[ref36] Chen C.; Zong S.; Wang Z.; Lu J.; Zhu D.; Zhang Y.; Cui Y. Imaging and Intracellular Tracking of Cancer-Derived Exosomes Using Single-Molecule Localization-Based Super-Resolution Microscope. ACS Appl. Mater. Interfaces 2016, 8 (39), 25825–25833. 10.1021/acsami.6b09442. 27617891

[ref37] Laney D. E.; Garcia R. A.; Parsons S. M.; Hansma H. G. Changes in the Elastic Properties of Cholinergic Synaptic Vesicles as Measured by Atomic Force Microscopy. Biophys. J. 1997, 72, 806–813. 10.1016/s0006-3495(97)78714-9. 9017205 PMC1185603

[ref38] Liang X.; Mao G.; Simon Ng K. Y. Probing Small Unilamellar EggPC Vesicles on Mica Surface by Atomic Force Microscopy. Colloids Surf., B 2004, 34 (1), 41–51. 10.1016/j.colsurfb.2003.10.017. 15261089

[ref39] Sharma S.; Gillespie B. M.; Palanisamy V.; Gimzewski J. K. Quantitative Nanostructural and Single-Molecule Force Spectroscopy Biomolecular Analysis of Human-Saliva-Derived Exosomes. Langmuir 2011, 27 (23), 14394–14400. 10.1021/la2038763. 22017459 PMC3235036

[ref40] Busatto S.; Yang Y.; Walker S. A.; Davidovich I.; Lin W.-H.; Lewis-Tuffin L.; Anastasiadis P. Z.; Sarkaria J.; Talmon Y.; Wurtz G.; Wolfram J. Brain Metastases-Derived Extracellular Vesicles Induce Binding and Aggregation of Low-Density Lipoprotein. J. Nanobiotechnol. 2020, 18 (1), 162 10.1186/s12951-020-00722-2. PMC7648399 33160390

[ref41] Priglinger E.; Strasser J.; Buchroithner B.; Weber F.; Wolbank S.; Auer D.; Grasmann E.; Arzt C.; Sivun D.; Grillari J.; Jacak J.; Preiner J.; Gimona M. Label-Free Characterization of an Extracellular Vesicle-Based Therapeutic. Journal of Extracellular Vesicles 2021, 10 (12), e12156 10.1002/jev2.12156. 34669269 PMC8528092

[ref42] Chiang C.; Chen C. Toward Characterizing Extracellular Vesicles at a Single-Particle Level. J. Biomed Sci. 2019, 26, 9 10.1186/s12929-019-0502-4. 30646920 PMC6332877

[ref43] Cavallaro S.; Pevere F.; Stridfeldt F.; Görgens A.; Paba C.; Sahu S. S.; Mamand D. R.; Gupta D.; El Andaloussi S.; Linnros J.; Dev A. Multiparametric Profiling of Single Nanoscale Extracellular Vesicles by Combined Atomic Force and Fluorescence Microscopy: Correlation and Heterogeneity in Their Molecular and Biophysical Features. Small 2021, 17 (14), 2008155 10.1002/smll.202008155. 33682363

[ref44] Strohmeier K.; Hofmann M.; Hauser F.; Sivun D.; Puthukodan S.; Karner A.; Sandner G.; Le Renard P.-E.; Jacak J.; Mairhofer M. CRISPR/Cas9 Genome Editing vs. Over-Expression for Fluorescent Extracellular Vesicle-Labeling: A Quantitative Analysis. International Journal of Molecular Sciences 2022, 23 (1), 282 10.3390/ijms23010282. PMC8745383 35008709

[ref45] Franke C.; Repnik U.; Segeletz S.; Brouilly N.; Kalaidzidis Y.; Verbavatz J.-M.; Zerial M. Correlative Single-Molecule Localization Microscopy and Electron Tomography Reveals Endosome Nanoscale Domains. Traffic 2019, 20 (8), 601–617. 10.1111/tra.12671. 31206952 PMC6771687

[ref46] Rennick J. J.; Johnston A. P. R.; Parton R. G. Key Principles and Methods for Studying the Endocytosis of Biological and Nanoparticle Therapeutics. Nat. Nanotechnol. 2021, 16 (3), 266–276. 10.1038/s41565-021-00858-8. 33712737

[ref47] Huang B.; Wang W.; Bates M.; Zhuang X. Three-Dimensional Super-Resolution Imaging by Stochastic Optical Reconstruction Microscopy. Science 2008, 319 (5864), 810–813. 10.1126/science.1153529. 18174397 PMC2633023

[ref48] Mayr S.; Hauser F.; Puthukodan S.; Axmann M.; Gohring J.; Jacak J. Statistical Analysis of 3D Localisation Microscopy Images for Quantification of Membrane Protein Distributions in a Platelet Clot Model. PLoS Comput. Biol. 2020, 16, e1007902 10.1371/journal.pcbi.1007902. 32603371 PMC7384682

[ref49] Sage D.; Pham T.-A.; Babcock H.; Lukes T.; Pengo T.; Chao J.; Velmurugan R.; Herbert A.; Agrawal A.; Colabrese S.; Wheeler A.; Archetti A.; Rieger B.; Ober R.; Hagen G. M.; Sibarita J.-B.; Ries J.; Henriques R.; Unser M.; Holden S. Super-Resolution Fight Club: Assessment of 2D and 3D Single-Molecule Localization Microscopy Software. Nat. Methods 2019, 16 (5), 387–395. 10.1038/s41592-019-0364-4. 30962624 PMC6684258

[ref50] Spotty. https://bioinformatics.fh-hagenberg.at/bin_typo3/htdocs/fileadmin/user_upload/Downloads/spotty.html.

[ref51] Allan D.; van der Wel C.; Keim N.; Caswell T. A.; Wieker D.; Verweij R.; Reid C.; soft-matter/trackpy: Trackpy, v0.4.2; 2019. 10.5281/zenodo.3492186.

[ref52] Tarantino N.; Tinevez J. Y.; Crowell E. F.; Boisson B.; Henriques R.; Mhlanga M.; Agou F.; Israël A.; Laplantine E. Tnf and Il-1 Exhibit Distinct Ubiquitin Requirements for Inducing NEMO-IKK Supramolecular Structures. J. Cell Biol. 2014, 204 (2), 231–245. 10.1083/jcb.201307172. 24446482 PMC3897181

[ref53] Wiesbauer M.; Wollhofen R.; Vasic B.; Schilcher K.; Jacak J.; Klar T. A. Nano-Anchors with Single Protein Capacity Produced with STED Lithography. Nano Lett. 2013, 13 (11), 5672–5678. 10.1021/nl4033523. 24111646

[ref54] Nečas D.; Klapetek P. Gwyddion: An Open-Source Software for SPM Data Analysis. Central European Journal of Physics 2012, 10 (1), 181–188. 10.2478/s11534-011-0096-2.

[ref55] Théry C.; Bussolati B.; Byrd J. B.; Carter D. R.; Chang Y.-T.; Chen S.; Chin A. R.; Clerici S. P.; Cocks A.; Coffey R. J.; Couch Y.; Coyle B.; Criado M. F.; Das S.; Candia P. de; Wever O. D.; Demaret T.; Devitt A.; Vizio D. D.; Dolo V.; Rubio A. P. D.; Dourado M. R.; Duarte F. V.; Eichenberger R. M.; Andaloussi S. E.; Erdbrügger U.; Fatima F.; Flores-Bellver M.; Frelet-Barrand A.; Fuhrmann G.; Gabrielsson S.; Gámez-Valero A.; Gardiner C.; Gärtner K.; Gaudin R.; Gho Y. S.; Giebel B.; Gilbert C.; Gimona M.; Giusti I.; Goberdhan D. C.; Görgens A.; Gorski S. M.; Greening D. W.; Gross J. C.; Gualerzi A.; Gupta G. N.; Gustafson D.; Handberg A.; Haraszti R. A.; Harrison P.; Hegyesi H.; Hendrix A.; Hill A. F.; Hochberg F. H.; Hoffmann K. F.; Holder B.; Holthofer H.; Hosseinkhani B.; Hu G.; Huang Y.; Huber V.; Hunt S.; Ibrahim A. G.-E.; Ikezu T.; Inal J. M.; Isin M.; Ivanova A.; Jackson H. K.; Jacobsen S.; Jay S. M.; Jayachandran M.; Jenster G.; Jiang L.; Johnson S. M.; Jones J. C.; Jong A.; Jovanovic-Talisman T.; Jung S.; Kalluri R.; Kano S.; Kaur S.; Kawamura Y.; Keller E. T.; Khamari D.; Khomyakova E.; Khvorova A.; Kierulf P.; Kim K. P.; Kislinger T.; Klingeborn M.; Klinke D. J.; Kornek M.; Kosanović M. M.; Kovács Á. F.; Krämer-Albers E.-M.; Krasemann S.; Krause M.; Kurochkin I. V.; Kusuma G. D.; Kuypers S.; Laitinen S.; Langevin S. M.; Languino L. R.; Lannigan J.; Lässer C.; Laurent L. C.; Lavieu G.; Lázaro-Ibáñez E.; Lay S. L.; Lee M.-S.; Lee Y. X. F.; Lemos D. S.; Lenassi M.; Leszczynska A.; Li I. T.; Liao K.; Libregts S. F.; Ligeti E.; Lim R.; Lim S. K.; Line̅ A.; Linnemannstöns K.; Llorente A.; Lombard C. A.; Lörincz Á. M.; Lötvall J.; Lovett J.; Lowry M. C.; Loyer X.; Lu Q.; Lukomska B.; Lunavat T. R.; Maas S. L.; Malhi H.; Marcilla A.; Mariani J.; Mariscal J.; Martens-Uzunova E. S.; Martin-Jaular L.; Martinez M. C.; Martins V. R.; Mathieu M.; Mathivanan S.; Maugeri M.; McGinnis L. K.; McVey M. J.; Meckes D. G.; Meehan K. L.; Mertens I.; Minciacchi V. R.; Möller A.; Jo̷rgensen M. M.; Morales-Kastresana A.; Morhayim J.; Mullier F.; Muraca M.; Musante L.; Mussack V.; Muth D. C.; Myburgh K. H.; Najrana T.; Nawaz M.; Nazarenko I.; Nejsum P.; Neri C.; Neri T.; Nieuwland R.; Nimrichter L.; Nolan J. P.; Hoen E. N. N.-’t; Hooten N. N.; O’Driscoll L.; O’Grady T.; O’Loghlen A.; Ochiya T.; Olivier M.; Ortiz A.; Ortiz L. A.; Osteikoetxea X.; Østergaard O.; Ostrowski M.; Park J.; Pegtel D. M.; Peinado H.; Perut F.; Pfaffl M. W.; Phinney D. G.; Pieters B. C.; Pink R. C.; Pisetsky D. S.; Strandmann E. P. von; Polakovicova I.; Poon I. K.; Powell B. H.; Prada I.; Pulliam L.; Quesenberry P.; Radeghieri A.; Raimondo S.; Rak J.; Ramirez M. I.; Raposo G.; Rayyan M. S.; Regev-Rudzki N.; Ricklefs F. L.; Robbins P. D.; Roberts D. D.; Rodrigues S. C.; Rohde E.; Rome S.; Rouschop K. M.; Rughetti A.; Russell A. E.; Saá P.; Sahoo S.; Salas-Huenuleo E.; Sánchez C.; Saugstad J. A.; Saul M. J.; Schiffelers R. M.; Schneider R.; Scho̷yen T. H.; Scott A.; Shahaj E.; Sharma S.; Shatnyeva O.; Shekari F.; Shelke G. V.; Shetty A. K.; Shiba K.; Siljander P. R.-M.; Silva A. M.; Skowronek A.; Snyder O. L.; Soares R. P.; Sódar B. W.; Soekmadji C.; Sotillo J.; Stahl P. D.; Stoorvogel W.; Stott S. L.; Strasser E. F.; Swift S.; Tahara H.; Tewari M.; Timms K.; Tiwari S.; Tixeira R.; Tkach M.; Toh W. S.; Tomasini R.; Torrecilhas A. C.; Tosar J. P.; Toxavidis V.; Urbanelli L.; Vader P.; Balkom B. W. van; Grein S. G. van der; Deun J. V.; Herwijnen M. J. van; Keuren-Jensen K. V.; Niel G. van; Royen M. E. van; Wijnen A. J. van; Vasconcelos M. H.; Vechetti I. J.; Veit T. D.; Vella L. J.; Velot É.; Verweij F. J.; Vestad B.; Viñas J. L.; Visnovitz T.; Vukman K. V.; Wahlgren J.; Watson D. C.; Wauben M. H.; Weaver A.; Webber J. P.; Weber V.; Wehman A. M.; Weiss D. J.; Welsh J. A.; Wendt S.; Wheelock A. M.; Wiener Z.; Witte L.; Wolfram J.; Xagorari A.; Xander P.; Xu J.; Yan X.; Yáñez-Mó M.; Yin H.; Yuana Y.; Zappulli V.; Zarubova J.; Žėkas V.; Zhang J.; Zhao Z.; Zheng L.; Zheutlin A. R.; Zickler A. M.; Zimmermann P.; Zivkovic A. M.; Zocco D. Minimal Information for Studies of Extracellular Vesicles 2018 (MISEV2018): A Position Statement of the International Society for Extracellular Vesicles and Update of the MISEV2014 Guidelines. Journal of Extracellular Vesicles 2018, 7 (1), 1535750 10.1080/20013078.2018.1535750. 30637094 PMC6322352

[ref56] Wolfesberger C.; Wollhofen R.; Buchegger B.; Jacak J.; Klar T. A. Streptavidin Functionalized Polymer Nanodots Fabricated by Visible Light Lithography. J. Nanobiotechnol. 2015, 13 (1), 27 10.1186/s12951-015-0084-6. PMC4453224 25888763

[ref57] McGuire H.; Aurousseau M. R. P.; Bowie D.; Bluncks R. Automating Single Subunit Counting of Membrane Proteins in Mammalian Cells. J. Biol. Chem. 2012, 287 (43), 35912–35921. 10.1074/jbc.M112.402057. 22930752 PMC3476259

[ref58] Chen Y.; Deffenbaugh N. C.; Anderson C. T.; Hancock W. O. Molecular Counting by Photobleaching in Protein Complexes with Many Subunits: Best Practices and Application to the Cellulose Synthesis Complex. Mol. Biol. Cell 2014, 25 (22), 3630–3642. 10.1091/mbc.e14-06-1146. 25232006 PMC4230622

[ref59] Coffman V. C.; Wu J. Q. Every Laboratory with a Fluorescence Microscope Should Consider Counting Molecules. Mol. Biol. Cell 2014, 25 (10), 1545–1548. 10.1091/mbc.e13-05-0249. 24825827 PMC4019486

[ref60] Liesche C.; Grußmayer K. S.; Ludwig M.; Wörz S.; Rohr K.; Herten D. P.; Beaudouin J.; Eils R. Automated Analysis of Single-Molecule Photobleaching Data by Statistical Modeling of Spot Populations. Biophys. J. 2015, 109 (11), 2352–2362. 10.1016/j.bpj.2015.10.035. 26636946 PMC4675885

[ref61] Dobrucki J. W.; Kubitscheck U. Determining Absolute Protein Numbers by Quantitative Fluorescence Microscopy Jolien. In Fluorescence Microscopy: From Principles to Biological Applications, 2nd ed.; 2017; pp 85–132.

[ref62] Dresser L.; Hunter P.; Yendybayeva F.; Hargreaves A. L.; Howard J. A. L.; Evans G. J. O.; Leake M. C.; Quinn S. D. Amyloid-β Oligomerization Monitored by Single-Molecule Stepwise Photobleaching. Methods 2021, 193 (June), 80–95. 10.1016/j.ymeth.2020.06.007. 32544592 PMC8336786

[ref63] Rautaniemi K.; John T.; Richter M.; Huck B. C.; Zini J.; Loretz B.; Lehr C.-M.; Vuorimaa-Laukkanen E.; Lisitsyna E.; Laaksonen T. Intracellular Dynamics of Extracellular Vesicles by Segmented Trajectory Analysis. Anal. Chem. 2022, 94 (51), 17770–17778. 10.1021/acs.analchem.2c02928. 36512439 PMC9798377

[ref64] Sako Y.; Kusumi A. Compartmentalized Structure of the Plasma Membrane for Receptor Move-ments as Revealed by a Nanometer-Level Motion Analysis. J. Cell Biol. 1994, 125 (6), 1251–1264. 10.1083/jcb.125.6.1251. 8207056 PMC2290914

[ref65] Day C. A.; Kenworthy A. K. Tracking Microdomain Dynamics in Cell Membranes. Biochimica et Biophysica Acta (BBA) - Biomembranes 2009, 1788 (1), 245–253. 10.1016/j.bbamem.2008.10.024. 19041847 PMC2792115

[ref66] Mattila P. K.; Feest C.; Depoil D.; Treanor B.; Montaner B.; Otipoby K. L.; Carter R.; Justement L. B.; Bruckbauer A.; Batista F. D. The Actin and Tetraspanin Networks Organize Receptor Nanoclusters to Regulate B Cell Receptor-Mediated Signaling. Immunity 2013, 38 (3), 461–474. 10.1016/j.immuni.2012.11.019. 23499492

[ref67] Poon C. Measuring the Density and Viscosity of Culture Media for Optimized Computational Fluid Dynamics Analysis of in Vitro Devices. Journal of the Mechanical Behavior of Biomedical Materials 2022, 126, 105024 10.1016/j.jmbbm.2021.105024. 34911025

[ref68] Schütz G. J.; Axmann M.; Schindler H. Imaging Single Molecules in Three Dimensions. Single Molecules 2001, 2 (2), 69–74. 10.1002/1438-5171(200107)2:2<69::AID-SIMO69>3.0.CO;2-N.

[ref69] Saint-Pol J.; Gosselet F.; Duban-Deweer S.; Pottiez G.; Karamanos Y. Targeting and Crossing the Blood-Brain Barrier with Extracellular Vesicles. Cells 2020, 9 (4), 851 10.3390/cells9040851. 32244730 PMC7226770

[ref70] Mayle K. M.; Le A. M.; Kamei D. T. The Intracellular Trafficking Pathway of Transferrin. Biochim. Biophys. Acta 2011, 1820 10.1016/j.bbagen.2011.09.009. PMC3288267 21968002

