# Supplementary material for: Purification Analysis, Intracellular Tracking, and Colocalization of Extracellular Vesicles Using Atomic Force and 3D Single-Molecule Localization Microscopy
Source: Anal Chem. 2023 Mar 31;95(14):6061–70. doi: 10.1021/acs.analchem.3c00144 (PMC10100414; doi:10.1021/acs.analchem.3c00144)
Supplement: Supplementary file 1 — ac3c00144_si_001.pdf [file ac3c00144_si_001.pdf]

Supporting information

Purification analysis, intracellular tracking and co-localization of extracellular vesicles using atomic force- and 3D single-molecule localization microscopy

Sujitha Puthukodan, Martina Hofmann, Mario Mairhofer, Hannah Janout, Jonas Schurr, Fabian Hauser, Christoph Naderer, Johannes Preiner, Stephan Winkler, Dmitry Sivun, Jaroslaw Jacak*

Sujitha Puthukodan - University of Applied Sciences Upper Austria, Linz 4020, Austria

Martina Hofmann - University of Applied Sciences Upper Austria, Linz 4020, Austria

Mario Mairhofer - University of Applied Sciences Upper Austria, Linz 4020, Austria

Hannah Janout – 1) University of Applied Sciences Upper Austria, Hagenberg 4232, Austria, 2) Johannes Kepler University, Department of Computer Science, Linz 4040, Austria

Jonas Schurr – 1) University of Applied Sciences Upper Austria, Hagenberg 4232, Austria, 2) Johannes Kepler University, Department of Computer Science, Linz 4040, Austria

Fabian Hauser - University of Applied Sciences Upper Austria, Linz 4020, Austria

Christoph Naderer - University of Applied Sciences Upper Austria, Linz 4020, Austria

Johannes Preiner - University of Applied Sciences Upper Austria, Linz 4020, Austria

Stephan Winkler – 1) University of Applied Sciences Upper Austria, Hagenberg 4232, Austria, 2) Johannes Kepler University, Department of Computer Science, Linz 4040, Austria

Dmitry Sivun - University of Applied Sciences Upper Austria, Linz 4020, Austria

Jaroslaw Jacak - University of Applied Sciences Upper Austria, Linz 4020, Austria

*Corresponding Author

Table of Contents

Additional information about AFM/FM image co-localization S2

Example of Single-molecule stepwise photobleaching analysis S2

Determination of cell membrane axial position S3

Stepwise Photobleaching software platform Spotty S4

Simulation of the of two-color correction S6

3D single-molecule two-color colocalization analysis..S8

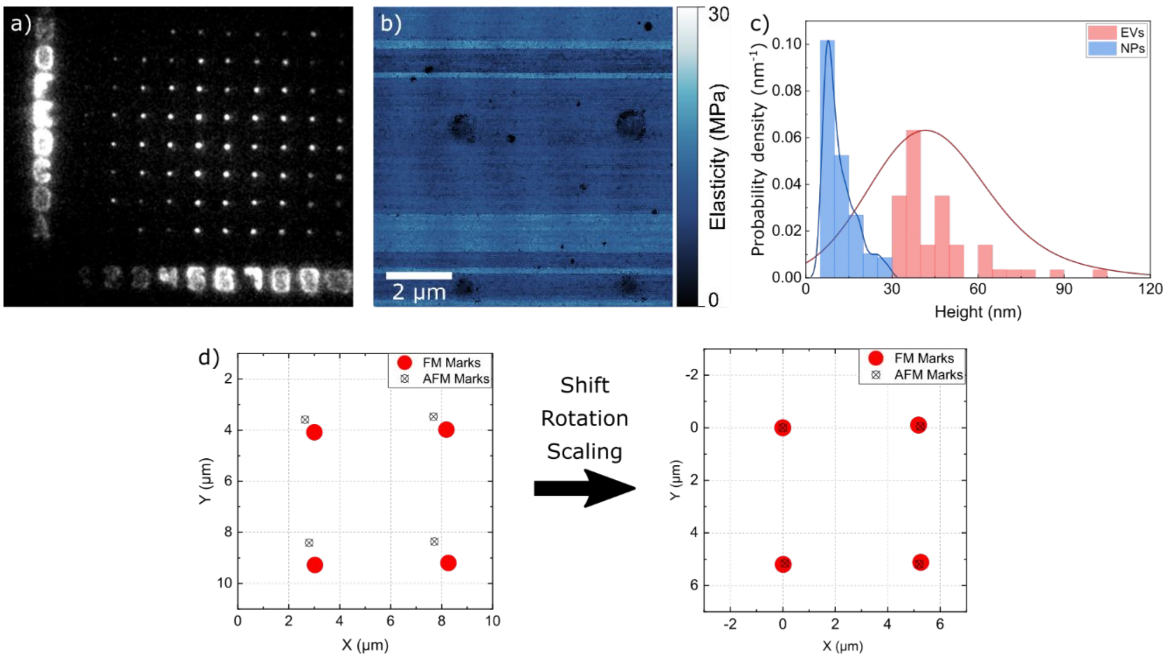

Figure SI 1 a) Fluorescence image of the marks grid used for co-localization of AFM and FM images. The image is taken in the red channel (Ex: 640 nm, Em: 700 nm). **b)** Young's modulus map of the area shown in Figure 1a). Only EVs (small black dots) are visible on top of the brighter background. **c)** Probability density distribution of the height of all particles detected in the AFM imaging. Two clearly separate peaks are visible (marked in red and blue colors) which we have corresponded to the EVs and other NPs respectively), **d)** Example of the FM image transformation needed to match the AFM data. The marks from the Figure 1 (a)-AFM and b)-FM are shown.

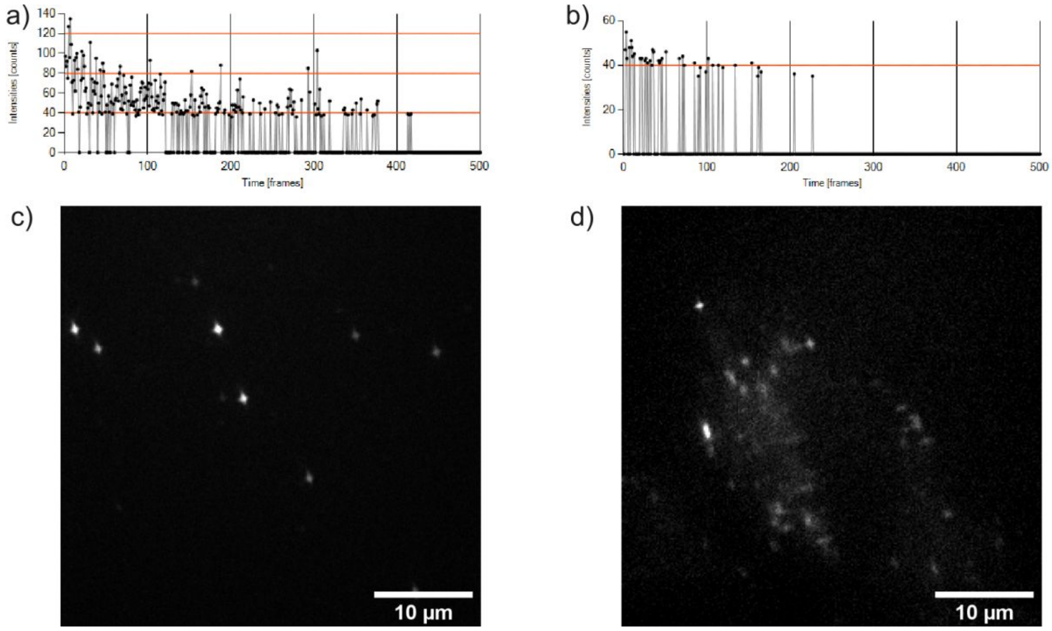

Figure SI 2 Single-molecule stepwise photobleaching. a) and **b)** shows the stepwise photobleaching tracks as function of time for EV on glass substrate and inside the cell, respectively. **c)** shows the fluorescent image of GFP CD63 EVs immobilized on glass substrate. **d)** shows the fluorescent image of fixed HeLa cells; the fluorescent signals correspond to the GFP CD63 EVs, which were internalized after incubation for ~30 min.

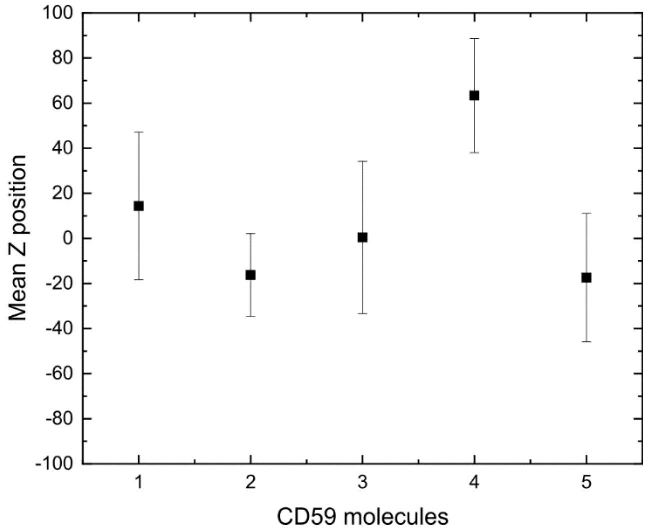

Figure SI 3 shows exemplary mean Z position (with standard deviation) of a CD59 molecule in cell membrane (averaged over full track length). It shows that CD59 has a confined movement on the membrane. The position accuracy is PA_{xy} = 42.2 nm and PA_z = 110.2 nm. The sequences were obtained at 10 ms illumination time, 12.3 fps and with a pixel size of 160 nm.

Stepwise Photobleaching software platform Spotty

The Spotty platform quantifies stepwise photobleaching of fluorescent molecule signals (e.g., sparsely distributed EVs with GFP-labelled CD63 proteins). Due to the partially low intensities of the single emitters and illumination irregularities of the images, homogeneous thresholding techniques are not feasible. A kernel filter is used for signal identification by considering the size and brightness of the signals. The kernel consists of a window of predefined size which scans over each image pixel-wise, while classifying each pixel value either as offset or signal. The pixels within the kernel are classified as background, sigma, and foreground. The foreground describes the inner area of the kernel, which defines the fluorescence signal area. The Background describes the area in the outer ring of the kernel, and sigma describes the ring-shaped area in-between the fore- and background used for spacing. The foreground- and background signal intensities are determined by calculating the averaged pixels' intensities of the areas. If the intensity difference between foreground and background is higher than a certain threshold and exceeds a minimum intensity, the current center pixel is counted as fluorescent signal. The analysis determines a set of individual fluorescence signals in each image within an image sequence. Next, the connection of the signals between the images is established. Tracking starts in the first image for each determined fluorescence signal.

Starting from the second image, the spatial distance between each signal to the previous image is computed. If the distance between the closest two fluorescence signals in two following images is below a user-defined threshold, they are associated with each other. A fluorescence signal, which could not be associated to a pre-existing track, will be regarded as a new track. Thus, a set of tracks is generated.

Typically, the signal intensity depends on the quantity of fluorescent emitters. Here, it is assumed that the signal of an average single emitter is constant (bleaching and inherent fluctuations disregarded). The intensities of multiple fluorescent molecules behave in an additive manner (quenching is disregarded), with their intensities only being influenced by noise, and illumination irregularities. Due to photobleaching within the image sequence, a temporal step-wise drop of the signal intensities is observable. Hence, analyzing this drop enables the determination of the intensity of a single emitter and the number of emitters per fluorescence signal. Due to these drops, the observed intensities of a signal can be clustered based on their similarity. For these clusters, the equidistant cluster centers are sought, which describe the data best. The intensity distance between clusters is equivalent to the intensity of a single emitter and referred to as delta.

To determine the value of a single emitter out of the time course of the photobleaching an exhaustive search is performed. In this search, with a step size of 1, all values starting from 1 up to the highest intensity of a tracked signal are set as delta. For each delta, equidistant centers are generated. Individual tracked signal intensities are assigned to the closest center. The quality of the resulting clusters is evaluated. The fit between cluster centers and the tracked signals clustered intensities is evaluated for each delta using the Davies Bouldin Index (DBI, a metric for the clustering quality). Given a total number of N cluster centers, the Davies Bouldin Index is defined as:

$$DBI(S, M) = \frac{1}{N} \sum_{i=1}^{N} \max_{i \neq j} \left(\frac{S_i + S_j}{M_{ij}} \right)$$

where S_i describes the dispersion of the respective clusters i and M_{ij} is defined as the separation between the clusters i and j. The dispersion S_i of cluster i is the maximum Euclidean distance of the cluster points to its center and the separation is the minimum distance between the centers of two clusters.

The DBI is always between zero and infinite. A lower number represents a better fit. The calculated value is based on the distance between the centers to their data points and the distance between the individual centers (estimated single emitter signals). The DBI aims to minimize the distance between centers and their data points, while maximizing the distance between individual clusters.

By evaluating the cluster fit with the DBI and choosing the delta with the lowest score, the best fitting centers for the signal's intensity data are determined. Thus, the intensity of a single emitter is equivalent to the found delta, and the total number of emitters is determined through the number of centers.

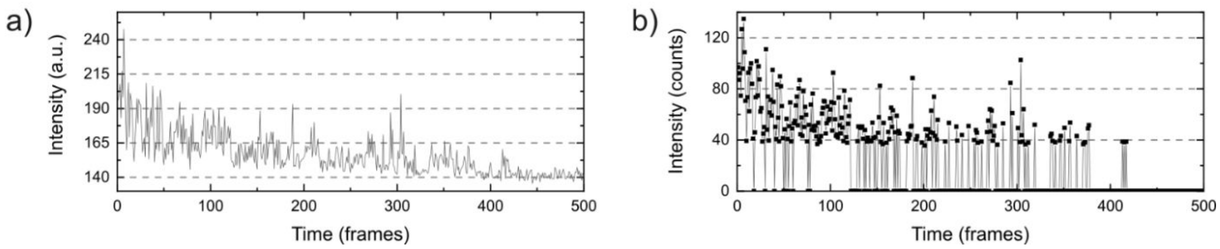

Figure SI 5 Single-molecule stepwise photobleaching. Comparison of raw signal intensity a) and intensity processed with the Spotty platform b).

Simulation of the of two-color correction

The chromatic dependent shift of single molecules from independent two color channels was simulated using real experimental values from [1] and [2]. We included both the "wobble" which shifts the lateral positions based on axial position and optical offsets based on the chromatic aberrations in our simulations. We simulated three different experiments, where we used either no wobble + low optical offsets, wobble + positive optical offsets and wobble + negative optical offsets. The offsets are calculated for central position (laser focus) on the focal plane and it is increased with increasing the distance from this central position (max offset 50 to 150 nm). Similar to the OptoSplit system, both color channels are within the same frame, split into two non-overlapping images. Using a registration, both channels can be overlayed again. The calibration contains frames at different axial positions with 10 nm steps over a range of 1 µm. The FWHM distortion (astigmatism) of single molecules dependent on the axial position was furthermore included. We used real-world calibration curves from 3D localization experiments for the different color channels (640 nm and 488 nm). Next, for testing we simulated circles of single molecules with decreasing radii as a function of the distance from the focal plane (z = 0 nm: r = 300 nm, z = ±150 nm: r = 200 nm, z = ±350 nm: r = 100 nm, z = ±450 nm: r = 0 nm). Within this test simulation only one channel contains the optical offset and because the list of single molecules were in the same arrangement, single molecule pairs from each channel were used to determine the accuracy of our correction.

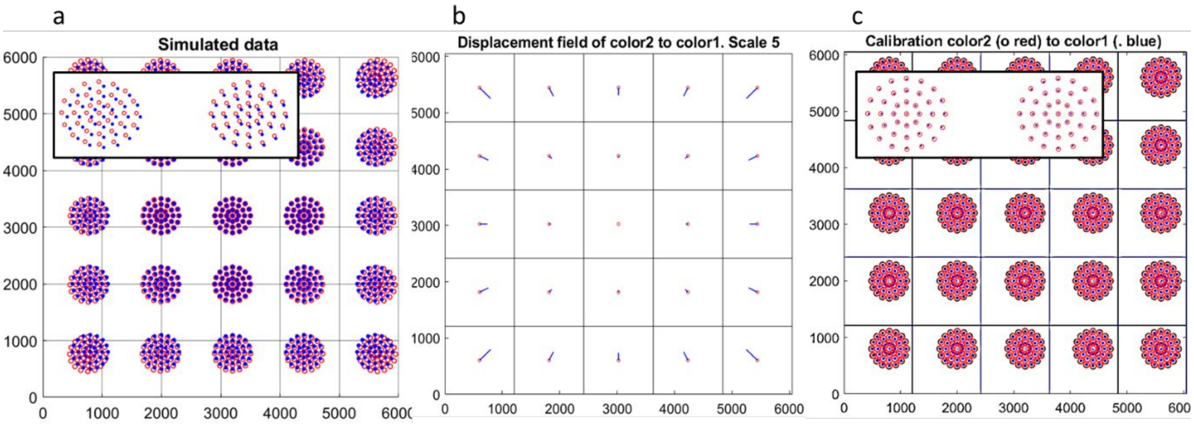

Figure SI 6 Two-color correction on simulated data a) Simulated localization data points with a positive wobble **b)** 3D Displacement vectors grid 5x5 for the color-correction. c**)** Simulated localization data after correction.

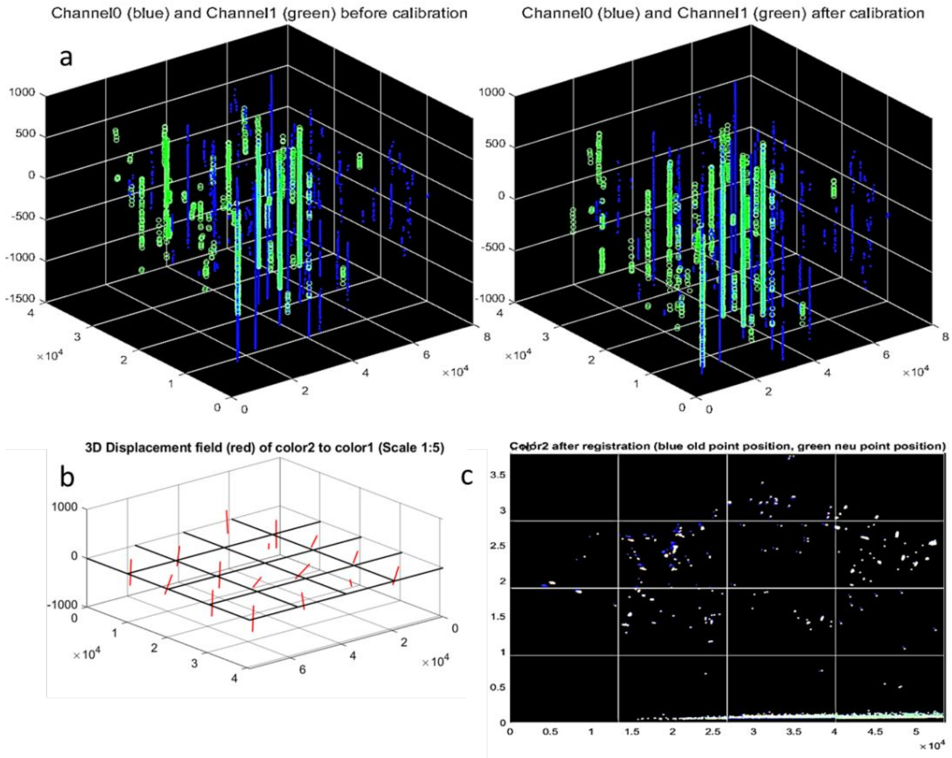

Figure SI 7 Two-color correction for cells a) displays the 3D transferrin Alexa 647 (blue) and the GFP CD63 EVs (green) localizations before and after the correction. **b)** 3D Displacement grid 4x4 for the color-correction. The grid displays a geometrical rigid transformation of channel 2 to channel 1. **c)** shows the GFP CD63 EVs 2D localizations before and after the correction. (scales in nm)

3D single-molecule two-color colocalization analysis

1 Calibration

The calibration is based on the geometric transformation of the image data of color channel 1 to the data of color channel 0. Images of both channels show fiducial markers which are necessary to calibrate the system for further experiments.

a) Fiducial marker points for calibration

Input data consists of fitted (3D STORM tools) fiducial fluorescent marker signals (tetraspeck bead signals[3,4]) originating from images of two channels: channel 0 called *fix point-cloud* and channel 1 *mobile point-cloud*. The fitted fiducial marker points of both color-channels are in one data set due to two-color imaging via OptoSplit[4]. It is important to cut out the points for each channel separately. Cutting is performed using ROI (Region Of Interest) parameters. The points from the ROI in color channel 1 will be moved relatively regarding the points within the ROI of the color channel 0 for correction of illumination heterogeneities and optical aberrations. First, brightness threshold and DBSCAN (Density-based spatial clustering of applications with noise) clustering[3] is used to combine position localizations into point-clouds (clusters) in both color channels. Points that do not belong to any cluster are removed.

An optimal rigid transformation (translation and rotation around the z-axis) will be sought for the localization of the points of color-channel 1 (moving points) relative to the localization of the points of color-channel 0 (fixed points). We assume a non-linear spatial shift between the color channels of the optical system; the optimal transformation computed for the full images averages the fluorescent signal shifts. However, such a transformation results in an inaccurate calibration at the image periphery (due to Gaussian illumination profile). For a better correction, we divided both sets of localized signals (images from both color channels) into sectors by overlaying a regular $n \times n$ grid. Our method is a generalization of the field displacement method typically used for image registration.[5,6] For each grid sector, the optimal rigid transformation of the sector's mobile points is calculated individually. The optimal transformation for each sector results from minimizing the sum of the minimum distances between mobile and fixed points. This results in an $n \times n$ displacement vector field representing the transformation for each grid sector separately. In some cases, fiducials are not densely distributed in the images, so increasing the size of the grid can result in a blank sector not containing localizations. To overcome this limitation, the transformation for these blank sectors is approximated to be the transformation point within the whole image (grid size =1). Small grid sizes with n from 2 to 7 are recommended to avoid a large number of empty sectors. The grid including displacement vectors is stored and can be used for registration and calibration of other localization points from two-color images.

2 Registration

Applying stored transformations in the form of a displacement vector grid to other samples with point localization data collected from channel 0 and channel 1 executes registration. Firstly, the data sets are assigned to a set of fixed points (fixed cloud) and a set of mobile points (mobile cloud). Mobile points are thus divided into sectors, defined by a displacement grid. The points within each sector are transformed locally according to the sector's displacement vector. Last, the shifted points of the mobile cloud are stored.

3 Colocalization

In case two samples contain point clouds in the same image region, co-localization is defined by the proximity of points in both samples within that region. Due to the high accuracy of the localized signals in both color channels, it is very probable that the intersection of the two-point clouds is empty. The co-localization challenge is to find groups of points in both samples overlapping each other. To find this overlap, we apply sensitive search.

4 Sensitive search

The initial step is to assign both point clouds of two-color localizations: the first as the fixed-point cloud and the second as the mobile point cloud. It is recommended to select as the mobile point cloud the sample with the smaller cloud size to speed up the analysis. Next, a union of mobile and fixed-point clouds is generated. This new set of points is clustered using the DBSCAN algorithm. The precision and quality of co-localization depends on the selection of DBSCAN parameters (i.e. radius R as threshold for a neighbourhood search and a minimum number of neighbours *minpts).* The value of *minpts* needs to be at least 4 to enable 3D convex hull constructs. The radius R is determined as a multiple of the average minimum distance between the point in new large cloud (i.e. *R* = *alpha* * *average_min_distance*). The selection of the radius *R* via the *alpha* factor is critical for the quality of the detected co-localization. Thus, the quality of the co-localization calculation finally depends on the determined alpha coefficient. For an accurate co-localization classification, alpha should be between 1 and 2. A search for clusters containing points from both clouds is performed after clustering, forming co-localization clusters. These co-localization clusters include points from two sub-clusters, the mobile cloud points (i.e. mobile sub-cluster) and the fixed cloud points (i.e. fixed sub-cluster). For co-localization clusters, the convex hulls for points originating from the mobile and fixed-point cloud and their volumes are calculated separately. If the convex hulls calculated this way have a non-empty intersection, then IoU is determined as the volume of this intersection in relation to the volume of both convex hulls. If the intersection is empty, the smallest distance (= *d_min*) between the points of the mobile sub-cluster and the points of the fixed sub-cluster is analyzed. For both analysed sub-clusters, the average position accuracy (mean of all dimensions) is calculated as *pa_mobile* and *pa_fixed*, respectively. In case the minimum distance between the points of both sub-clusters was zero, consequently the position localization inaccuracy distance is between zero and their sum *pa_mobile* + *pa_fixed*. Accordingly, non-intersecting sub-clusters with a minimum distance between their points < *pa_mobile* + *pa_fixed* can be regarded as very closely spaced. In this case, we decided to make the co-localization cluster size dependent as sometimes one of or both clusters is/are small (e.g., 2 points without a convex hull) and lack on intersection. Therefore, we determine co-localization only if both analyzed clusters have at least 3 points. IoU coefficient is calculated as the volume of the smaller of convex hulls in relation to the volume of both hulls.

After the co-localization is performed, four main parameters are calculated:

- volumetric average co-localization for sensitive method (IoU) as the quotient of the sum of the volumes of all intersecting/smaller parts of the clusters and the sum of the volumes of the whole co-localization clusters of the mobile and fix point cloud
- quantitative average co-localization for sensitive method: the quotient of the number of mobile point clouds within co-localization clusters and the number of all points within the mobile point cloud
- the distribution of the minimum distances between the points within the clusters of the mobile point-cloud and the points of fixed point-cloud (closest neighbors clusters)
- Histogram of the distribution of these distances.

References:

(1) Carlini, L.; Holden, S. J.; Douglass, K. M.; Manley, S. Correction of a Depth-Dependent Lateral Distortion in 3D Super-Resolution Imaging. *PLOS ONE* **2015**, *10* (11), e0142949. https://doi.org/10.1371/journal.pone.0142949.

(2) Erdelyi, M.; Rees, E.; Metcalf, D.; Schierle, G. S. K.; Dudas, L.; Sinko, J.; Knight, A. E.; Kaminski, C. F. Correcting Chromatic Offset in Multicolor Super-Resolution Localization Microscopy. *Optics Express* **2013**, *21* (9), 10978. https://doi.org/10.1364/OE.21.010978.

(3) Mayr, S.; Hauser, F.; Puthukodan, S.; Axmann, M.; Gohring, J.; Jacak, J. *Statistical Analysis of 3D Localisation Microscopy Images for Quantification of Membrane Protein Distributions in a Platelet Clot Model*; 2020; Vol. 16. https://doi.org/10.1371/journal.pcbi.1007902.

(4) Hauser, F.; Hauser, F.; Jacak, J.; Jacak, J. Real-Time 3D Single-Molecule Localization Microscopy Analysis Using Lookup Tables. *Biomed. Opt. Express, BOE* **2021**, *12* (8), 4955–4968. https://doi.org/10.1364/BOE.424016.
(5) Thirion, J.-P. Image Matching as a Diffusion Process: An Analogy with Maxwell's Demons. *Medical Image Analysis* **1998**, *2* (3), 243–260. https://doi.org/10.1016/S1361-8415(98)80022-4.
(6) Vercauteren, T.; Pennec, X.; Perchant, A.; Ayache, N. Diffeomorphic Demons: Efficient Non-Parametric Image Registration. *NeuroImage* **2009**, *45* (1, Supplement 1), S61–S72. https://doi.org/10.1016/j.neuroimage.2008.10.040.
